# Supplementary material for: Comparative Proteomic Analysis of Proteins in Breast Milk during Different Lactation Periods
Source: Nutrients. 2022 Sep 3;14(17):3648. doi: 10.3390/nu14173648 (PMC9460426; doi:10.3390/nu14173648)
Supplement: Supplementary file 1 [file nutrients-14-03648-s001.zip › nutrients-1895447-supplementary.pdf]

nutrients

MDPI

Table S1. Information of participants

Mother	Age (Years)	Height (cm)	Weight (kg)	Parity	Gender of Baby	Birth Weight (g)	Birth Mode: Vaginal Delivery (V) or Cesarean Section (C)
Mother1	34	164	72	1	female	3550	V
Mother2	23	169	67	1	male	3800	C
Mother3	26	160	57	1	male	3300	V
Mother4	30	164	70	1	female	4300	C
Mother5	32	153	49	1	female	2500	V
Mother6	29	160	52	1	female	3500	C
Mother7	32	158	62	1	female	2950	V
Mother8	26	158	55	1	female	2350	V
Mother9	31	166	65	1	female	2900	V
Mother10	30	170	75	1	female	3450	V
Mother11	25	156	50	1	male	2650	V
Mother12	25	158	52	1	female	3050	C
Mother13	25	162	69	1	female	4200	C
Mother14	25	158	50	2	female	3500	V
Mother15	25	165	69	1	female	3850	V
Mother16	28	162	70	1	female	3200	V
Mother17	26	164	58	1	female	2800	V
Mother18	24	160	80	1	male	3100	V
